# Preachers and Subjects for Hospital Sunday

**Published:** 1888-06-09

**Authors:** 


### PREACHERS AND SUBJECTS FOR HOSPITAL SUNDAY.

The following is a list of preachers and subjects of sermons to be delivered in the respective places of worship on the 10th inst., on behalf of the Hospital Sunday Fund :—

All Saints' Church, Peckham.—Rev. T. J. Gaster.

Brentford Congregational Church.—11 a.m., Rev. W. Edwards.

Belgrave Presbyterian Church, W.—"The Disciples filled with Joy and the Holy Ghost."

Chapel of Guy's Hospital.—11 a.m., Rev. S. W. Gent, M.A., Principal of St. Mark's College, Chelsea; 7 p.m., Rev. John Griffiths, M.A., Christ Church, Battersea.

Christ Church, Lancaster Gate.—11 a.m., Rev. J. E. C. Welldon, M.A., Head Master of Harrow School; 3 p.m., Children's Flower Service, Rev. G. Ridgeway, vicar; 4.15 p.m., Rev. G. Ridgeway, vicar; 7.0 p.m., Rev. F. E. Ridgeway, M.A., incumbent of St. Mary's, Glasgow.

Church of Holy Cross in St. Pancras, Cromer Street, King's Cross.—Special sermons at the 11 a.m. and 7 p.m. services by Albert Moore, M.A., vicar (Church of England.)

Curzon Chapel.—11.30 a.m., Rev. Edward K. Gray, "The Tetrarch's Choice"; 6.30 p.m., "Dives and Lazarus."

Christ Church, Brondesbury.—Rev. C. W. Williams, D.D., 2 Kings xx. 5—7.

Christ Church, Greenwich.—Rev. Dr. Reith, "This World's Good."

Dulwich College Chapel.—11 a.m., the Rev. J. Dana; 7 p.m., the Rev. G. C. Allen; 5 p.m., Organ Recital and Collection for the Fund.

Dutch Church, Austinfriars, E.C.—Rev. A. D. Adama Van Scheltema, "To have all things in common is also to bear one another's burdens."

Eccleston Square Church, Belgrave Road, S.W.—Morning 11, Rev. J. Hiles Hitchens, D.D., F.S.Sc.

Essex Church, The Mall, Notting Hill Gate.—Rev. W. Carey Walters. 11 a.m., "The City of God"; 7 p.m., "Savonarola, the Reformer of Florence."

Emmanuel, Forest Gate, Parish Church.—11 a.m Right Rev. the Lord Bishop of N. Queensland; 3.15 p.m, (to the young.) Right Rev. the Lord Bishop of N. Queensland; 6.30 p.m., the Rev. R. Ross, vicar.

Holy Trinity Church, Lee, S.E.—Rev. B. W. Buckle, M.A., "Work for Christ in Connexion with Hospitals."

Holy Trinity Church, Upper Tooting.—Rev. J. Harlock Potter, 11 a.m., "Heal her now."

Holy Innocents' Church, Hornsey.—"Bear ye one another's burdens."

Hornsey Parish Church.—Rev. J. Jeakes, morning, "The Use of Pain."

Holy Trinity, Sydenham.—Rev. Henry Stevens, M.A., "What we are to others, an evidence of what we are ourselves, and a prediction of what we are to be in the worldly life to come."

Holy Trinity, Blackheath Hill.—11 a.m., Rev. J. G. Leeke; 7 p.m., Rev. S. F. Hooper, "Perfect through Suffering."

Hampstead Parish Church.—Rev. S. B. Burnaby, "Christian Brotherhood."

Islington Presbyterian Church, Colebrook Row, N.—11 a.m., Rev. Thain Davidson, D.D., "The Duty of Discriminate Charity."

Marsh Street Congregational Church, Walthamstow.—Rev. S. Conway, B.A.

Parish Church, Wandsworth; Holy Trinity Church, Wandsworth; St. Michael's Church, Wandsworth. Preachers not stated.

Parish Church, Stoke Newington, N.—Rev. J. Robinson, "Christ Healing the Sick."

Park Chapel, Camden Town.—Rev. Joshua C. Harrison, "Sorrow turned into joy" (John xvi. 20).

Rosslyn Hill Chapel, Hampstead.—Rev. Dr. Sadler, "The Blessing of Health."

St. Andrew's, Thornhill Square, N.—Rev. A. J. Bridgman.

St. Augustine's, Honor Oak Park.—11 a.m. and 7 p.m., Vicar, "Receivers and Givers."

St. Bartholomew's Church, Sydenham.—Rev. Canon Evan Daniel, "Christianity in its Relation to Human Suffering."

St. Giles's, Camberwell.—11 a.m., Rev. F. F. Kelly; 7 p.m., Dean of Rochester.

St. Gabriel's, Warwick Square.—7 p.m., the Rev. A. Williamson.

St. George's-in-the-East.—Rev. William Sinclair, evening, "The Touch of the Hand of God."

St. John, Horselydown, S.E.—6.30 p.m., Rev. W. J. Batchelor, rector.

St. James, Knatchbull Road, Camberwell.—11 a.m., Rev. J. D. Dykes, vicar, "She hath done what she could"; 7 p.m., Rev. J. C. Lintott, vicar of St. Philip's, Battersea.

St. James's, Norlands, Notting Hill.—11 a.m., the Rev. A. Williamson.

St. Jude's, South Kensington, S.W.—11 a.m., Rev. Prebendary Forrest, D.D.; 4 p.m., Flower Service; 7 p.m., Archdeacon Farrar.

St. John's Church, Clapham Rise, S.W.—Rev. C. H. C. Baker, D.D. — Morning, "God's Stewards." Afternoon, "Receiving and Giving." Evening, "My Neighbour."

St. Leonard's, Streatham.—Rev. J. R. Nicholl—Acts x. 38.

St. Mary's, Vincent Square.—Rev. J. MacArthur, "Our Lord's Work in Earth and in Heaven in its bearing on Sickness."

*(Continued on page 162.)*

### PREACHERS AND SUBJECTS FOR HOSPITAL SUNDAY.

(*Continued from p.* 158.)

St. Michael's, Paddington.—11.30 a.m., Rev. G. F. Prescott, "Brotherly Love."

St. Mark's, Notting Hill.—11 a.m. Rev. W. E. Emmett vicar—subject, "The Beatitude of Unselfishness."

St. Matthew's, Denmark Hill.—11 a.m., Rev. J. B. Pattrick, M.A.; 3 p.m., Rev. E. A. Wesley, M.A.; 7 p.m., Rev. Sinclair Carolin, M.A.

St. Martin's Church.—Rev. J. F. Kitto, "Benevolence the Natural Expression of Christianity."

St. Mary, Newington.—The Rector, "Willing Sacrifice the Characteristic of Christian Almsgiving."

St. Philip's, Regent Street, Waterloo Place.—11.15 a.m., Rev. Harry Jon es—subject, "Use and Abuse of Hospitals"; 4 p.m., Canon Duckworth—subject, "Medical Relief."

St. Peter's Church, Bayswater.—Rev. Charles Green, "Kind Deeds and Spotless Character the Best Ritual."

St. Peter's, Vere Street.—11 a.m., the Rev. W. Page Roberts, "Am I my Brother's Keeper?" 3 p.m., the Rev. G. L. Daniell, Children's Service.

St. Peter's, Lee.—Rev. R. J. Simpson, morning, Romans v. 12 and 1 Cor. xii. 25.

St. Peter's, Brockley, S.E.—Rev. G. F. Grundy; morning, "Uses of Pain;" evening, "Remedies for Pain."

St. Paul's, Clapham.—Rev. G. Forrester—Morning, "The Widow's Mite." Evening, "Sick nigh unto Death."

St. Stephen's, Southwark.—11 a.m., Rev. A. P. Robinson, M.A.; 6.30 p.m., Rev. W. Dodge, B.A.

St. Stephen's, Westminster.—Rev. William Sinclair, morning, "The Gift which Cost Nothing."

St. Saviour's, South Hampstead.—7 p.m., Rev. G. F. Prescott, "Sons of Consolation."

St. Stephen's, South Lambeth.—11 a.m. and 6.45 p.m., Vicar.

St. Saviour's, Denmark Park.—Rev. H. Swithinbank, "Love Looking Onwards."

St. Thomas's Church, Hemingford Road, Islington.—11 a.m. and 6.30 p.m., Rev. Edward Brewer, vicar.

Stoke Newington Old Church.—Rev. J. E. Shelford, "Charity Covering Sin."

Trinity Church, Hampstead.—Rev. H. Sharpe, "James yielded his life for Christ—what are we doing for the afflicted and the sorrowing to-day?"

Theistic Church, Swallow Street.—Rev. Charles Voysey, "Moral Uses of Pain."

Westminster Abbey.—The Rev. E. C. Dermer, "The Witness of Good Works in the Gospel."

Woodford, Essex.—Rev. W. E. Anderton, "The Saviour of the Body."

West Hackney Parish Church.—Morning, Rev. H. E. B. Arnold, M.A. Evening, Rev. L. H. Bradford.

